# Observational Molecular Case-Control Study of Genetic Polymorphisms 1 in Programmed Cell Death Protein-1 in Patients with Oral Lichen Planus

**DOI:** 10.31557/APJCP.2019.20.2.421

**Published:** 2019

**Authors:** Janan Ghapanchi, Hamid Ghaderi, Mohammad Reza Haghshenas, Samira Jamshidi, Fahimeh Rezazadeh, Azita Azad, Mitra Farzin, Reza Derafshi, Ahmad Hasan Kalantari

**Affiliations:** 1 *Department of Oral Medicine, *; 5 *Oral and Dental Disease Research Center, Department of Oral Medicine,*; 6 *Department of Prosthetic, School of Dentistry, *; 2 *Shiraz Institute for Cancer Research,*; 3 *Cancer Immunology Group, Shiraz Institute for Cancer Research, School of Medicine, Shiraz University of Medical Sciences, Shiraz,*; 4 *Department of Oral Medicine, School of Dentistry, Gorgan University of Medical Sciences, Gorgan *

**Keywords:** Oral lichen planus, single nucleotide polymorphism, programmed cell death protein, autoimmune disease

## Abstract

**Background:**

The association between programmed cell death protein 1 (PD-1) variations and susceptibility to autoimmune diseases has been recurrently reported. However, there is no report about its relationship with oral lichen planus (OLP) as one of autoimmune diseases.

**Methods:**

We investigated the association between genetic predisposition to OLP and two single nucleotide polymorphisms in PD-1.

**Results:**

GG, GA, and AA genotypes at position +7146 were found in 59 (80.8 %), 10 (13.7 %), and 4 (5.5 %) patients, and in 132 (77 %), 34 (20 %), and 5 (3 %) healthy participants. CC, CT, and TT genotypes at position +7785 were found in 32 (43.8 %), 35 (47.9 %), and 6 (8.2 %) patients and in 99 (58 %), 66 (39 %), and 6 (3 %) controls. Analysis indicated that patients’ genotypes were not statistically different from controls’ genotypes at both positions +7146 (P = 0.35 and P = 0.98) and +7785 (P = 0.07 and P = 0.06).

**Conclusion:**

The findings indicated that PD-1 SNPs at +7146 [PD-1.3] G/A and +7785 [PD-1.5] C/T was not associated with susceptibility to OLP. However, further research with higher sample size and in different geographical regions is needed in order to achieve the generalizability of the findings.

## Introduction

Oral lichen planus (OLP) is the most common chronic, non-infectious, autoimmune disease of the soft tissue, affecting 1-2 % of adult patients in oral medicine clinics (Ghapanchi et al., 2015). This disease most frequently manifests as lesions in the buccal mucosa (Axéll and Rundquist 1987; Sugerman, Savage et al., 2002; Rezazadeh et al., 2017) and may appear as bilateral, fiery red erythema across the entire width of the attached gingiva (Sugerman and Sabage, 2002; Lavaee et al., 2018). In addition, Some patients experience genital lesions with features similar to skin lesions .(Franciscoet al., 2010; Fahimeh et al., 2013). The World Health Organization (WHO) has defined OLP as a potentially malignant disorder, representing a generalized state associated with a significantly increased risk of cancer. Malignant transformation has been estimated to occur in 0.5-2.9% of the OLP patients. Currently, there are no prognostic markers to identify which chronic OLP lesions are at a higher risk for progression. Thus, every OLP patient should be monitored carefully to detect early cancer development. To understand the etiopathogenesis of OLP, it is important to recognize the key molecules in it. Molecular markers offer the possibility to identify patients with potentially malignant lesions which are in progression toward cancer before malignant cells are detectable histologically, at the primary site. 

In humans, binding between programmed cell death protein 1 (PD-1) and its ligand PD-L1 terminates the immune response. Therefore, deletion, mutation, and some polymorphisms in PD- 1 induce autoimmunity (Shinohara et al., 1994; Vibhakar et al., 1997; Ghapanchi et al., 2014). PD-L1 is expressed on activated CD4+ T cells, and it is a co-inhibitory signal that promotes apoptosis in activated T-cells (Yamamoto et al., 2008; Shi et al., 2013). PD-L1 expression is also upregulated in Hodgkin lymphoma cells, while PD-1 expression is markedly elevated in tumour infiltrating or peripheral T cells in patients (Yamamotoet al., 2008; Shi et al., 2013).

Despite the centrality of PD-1 in immunity and autoimmunity, its link to OLP has not been intensively examined. Thus, we investigated the association between genetic susceptibility to the disease and PD-1 SNPs at positions +7146 [PD-1.3] G/A and +7785 [PD- 1.5] C/T.

## Materials and Methods

*Patients*

The study protocol, based on a case-control design, was approved by the local ethics committee of Shiraz University of Medical Sciences, Shiraz, Iran, and it was in line with the World Medical Association Declaration of Helsinki (version, 2002). Written informed consent was obtained from all the patients. Blood samples were obtained from 104 patients with OLP, whose disease was confirmed through clinical examination and histopathological testing at the Oral Medicine Department, Shiraz University of Medical Sciences. Among 104 patients, 14 were male. Patients aged between 27 to 105 years old with mean age of 39 ± 13 years. There was no history of systemic disease, cancer, or other autoimmune diseases in these patients and in their first-degree relatives . In addition, patients had not received medications that might produce a lichenoid reaction within three months prior to blood draw. The control population consisted of 171 healthy subjects, 37 of whom were male, with mean age of 43 ± 14 years (19-65 years), and without family history of cancer or autoimmune disease. All the participants were residents of southern Iran.

*DNA extraction and genotyping*

DNA was extracted from peripheral white blood cells by salting out method (Mardani et al., 2012). Samples were genotyped at the PD-1.3 and PD-1.5 positions by polymerase chain reaction-restriction fragment length polymorphism (PCR-RFLP) and nested PCR-RFLP (Miller et al., 1988). Primers were synthesized by Takapouzist, Iran. Primer sequences, PCR parameters, and restriction enzymes used for each SNP are summarized in Table 1.

*Statistical analysis*

Genotype frequency was evaluated by Arlequin version 3.5.2, integrated software for population genetics data analysis, and analysed using SPSS (version 19). Groups were compared using Pearson’s χ^2^ test. Significance level was set at P < 0.05.

## Results

At position PD-1.3, the GG genotype was the most prevalent which was found in 79 patients (80.8 %), while the GA and AA genotypes were found in 10 (13.7 %) and 4 (5.5 %) patients, respectively (Table 2). Among control group, 132 (77 %), 34 (20 %), and 5 (3 %) were GG, GA, and AA genotypes; respectively. The distribution of genotypes was significant between patients and controls (P = 0.35). At position PD-1.5, 32 patients (43.8 %) were genotype CC, while 35 (47.9 %) and 6 patients (8.2 %) had 58 CT and TT genotype, respectively. In the control group, CC, CT, and TT genotypes were found in 99 (58 %), 66 (39 %), and 6 (3 %) individuals; respectively. The most prevalent genotype among patients was CT heterozygote, and among controls was CC homozygote. SNPs were not significantly different between two groups (P = 0.07). The most common allele found in both groups was the wild type C allele (P = 0.06). Significant relation was observed between sex and genetic polymorphism of the PD-1 gene among patients and its prevalence was significantly higher in female (p<0.05 ).

**Table1:** Specific Primers and Reaction Conditions for PD-1.3 and PD-1.5 Positions

Locus	Primer	Primer sequence	AT	RE	LDE
+7146 G/A [PD-1.3]	F	5-GCCTGGAGGACTCACATTCT-3	58 °C	PST I	G:381bp
	R	5-GTCCCCCTCTGAAATGTCC-3			A: 277bp, 104bp
+7785 C/T [PD-1.5]	F (outer reaction)	5'-AGACGGAGTATGCCACCATTGTC-3'	58 °C	PVU II	C: 89bp
	R (outer reaction)	5'-AAATGCGCTGACCCGGGCTCAT-3'			T: 48bp, 41bp
	F (inner reaction)	5'-TAGCGGAATGGGCACCTCATC-3'	51 °C		
	R (inner reaction)	5'-AGTGTCCATGCTCAGGCCTCA-3'			

**Table 2:** Genotypes and Allele Frequencies of PDCD-Lgene in Positions PD 1.3 and PD 1.5 in Patients with Oral Lichen Planus in Comparison to Controls

Locus	SNPs	Cases (N=73)	Controls (N=71)	P Value
+7146 G/A [PD-1.3]	GG	59 [80.8%]	132 [77%]	0.35
	GA	10 [13.7%]	34 [20%]	
	AA	4 [5.5%]	5 [3%]	
	Missing	-	-	
	G	128 [87%]	298 [87%]	0.98
	A	18 [13%]	44 [23%]	
+7785 C/T [PD-1.5]	CC	32 [43.8%]	99 [58%]	0.07
	CT	35 [47.9%]	66 [39%]	
	TT	6 [8.2%]	6 [3%]	
	Missing	-	-	
	C	99 [68%]	254 [76.5%]	0.06
	T	47 [32%]	78 [23.5%]	

231 SNPs, single, nucleotide polymorphisms.

## Discussion

In the present study, the relationship between genetic polymorphism of the PD-1 gene and predisposition to OLP was evaluated. No associations were found between +7146 [PD-1.3] G/A and +7785 [PD-1.5] C/T genetic polymorphism and susceptibility to OLP. OLP most frequently manifested in the buccal mucosa, and less frequently in the tongue and buccal mucosa, or in the tongue and gingiva (Yamamoto et al., 2008; Francisco et al., 2010; Shi et al., 2013). Many factors, including inflammatory cells, cytokines, and matrix metalloproteinases, can trigger disease development (Sugerman and Sabage, 2002). In particular, most studies have focused on proteins that regulate the immune system, as well as other factors that impact autoimmunity (Okazaki and Honjo, 2007; Neshat et al., 2013, Ghapanchi et al., 2014). One such factor is PD-1, a receptor that negatively regulates T-cell activation. PD-1 is expressed on activated T and B lymphocytes, and binding with its ligand blocks cellular changes induced by CD3/CD28 (Agata et al., 1996; Parry et al,. 2005]. PD-1 deficiency hyperactivates the immune system in mice and stimulates B cell proliferation in vitro (Nishimura et al., 1999). On the other hand, Thompson et al. found that patients with high levels of PD-L1 in renal cell tumors and/or lymphocytes were 4.5 times more likely to die from the carcinoma (Thompson et al., 2004).

The frequency of G/G and A/A genotypes in PD-L1 was significantly different between infertile patients and controls in Iran. Allele frequencies of PD-1 were also significantly different (Zamani et al., 2015). In a survey on 296 patients with various forms of cancer, Topalian et al. concluded that antibodies against PD-1 induced objectively measurable responses in 20-25 % of patients with non-small cell lung cancer, melanoma, or renal-cell cancer (Thompson et al., 2004; Topalian et al., 2012). Finally, Zandberg and Strome (2014) described the role of PD-L1 and PD-1 in head and neck squamous cell carcinoma, and carefully considered how their activities can be manipulated as a therapeutic strategy.

However, the association among oral lesions, PD-1, and other regulators of the immune response has not been examined in great detail. In a comparison of 105 unaffected subjects and 35 patients with oral lesions in Shiraz, Iran, Ghapanchi et al., (2014) did not find a significant association between the disease and polymorphisms at position +49 A/G in the CTLA-4, another co-inhibitor of the immune response. Similarly, polymorphisms in tumor protein p53 codon 72 did not correlate with susceptibility to OLP (Ghabanchi et al., 2009). In line with these results, we found no relevant correlation between the disease and PD-1 polymorphisms at positions PD-1.3 and PD-1.5. Nevertheless, several reports have identified possible biomarkers in the serum. For instance, Farzin et al. found that serum levels of matrix metalloproteinase 3 were significantly higher in patients than in healthy controls, and that variations in the clinical presentation of the disease was associated with significant differences in serum levels of the enzyme (Farzin et al., 2012).

In conclusion, the current research showed that the polymorphism of PD-1.3 and PD-1.5 genes did not have any significant correlation with OLP susceptibility. However, our study suffers from small number of participants in each group since we could not access to larger sample size and we enrolled all available patients with pathological diagnosis of OLP. Further studies are suggested with higher sample size and in different geographical regions in order to achieve the generalizability of the findings.

## Abbreviations

AT: annealing temperature; LDF: length of digested fragments; OLP: oral lichen planus; PD-1: programmed cell death protein-1; PD-L: PD-ligand; RE: Restriction enzyme; SNPs: single nucleotide polymorphisms.

## Competing Interests

The authors declare that they have no competing interests.
